# Bis{2-eth­oxy-6-[2-(isopropyl­ammonio)ethyl­imino­meth­yl]phenolato}dithio­cyanato­nickel(II)

**DOI:** 10.1107/S1600536809055780

**Published:** 2010-01-09

**Authors:** Chen-Yi Wang, Jin-Yun Ye, Xiang Wu, Cai-Jun Yuan

**Affiliations:** a Department of Chemistry, Huzhou University, Huzhou 313000, People’s Republic of China

## Abstract

In the mononuclear title complex, [Ni(NCS)_2_(C_14_H_22_N_2_O_2_)_2_], the Ni atom lies on an inversion centre. It is chelated by the phenolate O and imine N atoms from two zwitterionic Schiff base ligands, and is also coordinated by the N atoms from two thio­cyanate ligands, giving a slightly distorted octa­hedral geometry. Intra­molecular N—H⋯O and N—H⋯N hydrogen bonds are observed.

## Related literature

For related structures, see: Ali *et al.* (2004 ▶); Sarı *et al.* (2006 ▶); Gomes *et al.* (2000 ▶); Su *et al.* (2006 ▶); Wang (2007 ▶).
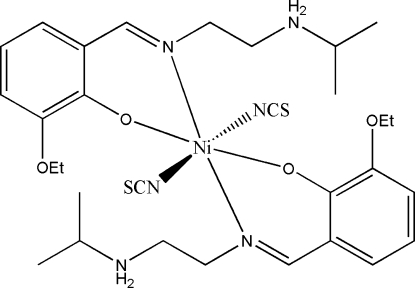


## Experimental

### 

#### Crystal data

[Ni(NCS)_2_(C_14_H_22_N_2_O_2_)_2_]
*M* *_r_* = 675.54
Monoclinic, 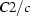

*a* = 24.958 (3) Å
*b* = 14.016 (2) Å
*c* = 9.613 (2) Å
β = 91.73 (2)°
*V* = 3361.2 (9) Å^3^
*Z* = 4
Mo *K*α radiation
μ = 0.74 mm^−1^
*T* = 298 K
0.32 × 0.30 × 0.30 mm

#### Data collection

Bruker SMART CCD area-detector diffractometer
Absorption correction: multi-scan (*SADABS*; Sheldrick, 1996 ▶) *T*_min_ = 0.797, *T*_max_ = 0.808
9655 measured reflections
3553 independent reflections
2395 reflections with *I* > 2σ(*I*)
*R*_int_ = 0.046

#### Refinement

*R*[*F* ^2^ > 2σ(*F* ^2^)] = 0.046
*wR*(*F* ^2^) = 0.115
*S* = 1.03
3553 reflections
199 parameters
H-atom parameters constrained
Δρ_max_ = 0.56 e Å^−3^
Δρ_min_ = −0.36 e Å^−3^

### 

Data collection: *SMART* (Bruker, 1998 ▶); cell refinement: *SAINT* (Bruker, 1998 ▶); data reduction: *SAINT*; program(s) used to solve structure: *SHELXS97* (Sheldrick, 2008 ▶); program(s) used to refine structure: *SHELXL97* (Sheldrick, 2008 ▶); molecular graphics: *SHELXTL* (Sheldrick, 2008 ▶); software used to prepare material for publication: *SHELXTL*.

## Supplementary Material

Crystal structure: contains datablocks global, I. DOI: 10.1107/S1600536809055780/ci5007sup1.cif

Structure factors: contains datablocks I. DOI: 10.1107/S1600536809055780/ci5007Isup2.hkl

Additional supplementary materials:  crystallographic information; 3D view; checkCIF report

## Figures and Tables

**Table 1:** Selected bond lengths (Å)

Ni1—O1	2.0104 (18)
Ni1—N1	2.076 (2)
Ni1—N3	2.180 (3)

**Table 2:** Hydrogen-bond geometry (Å, °)

*D*—H⋯*A*	*D*—H	H⋯*A*	*D*⋯*A*	*D*—H⋯*A*
N2—H2*B*⋯N3	0.90	2.34	3.113 (3)	144
N2—H2*A*⋯O2^i^	0.90	2.53	3.273 (3)	141
N2—H2*A*⋯O1^i^	0.90	1.79	2.584 (3)	145

Symmetry code: (i) 
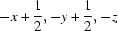
.

**Fig. 1.**
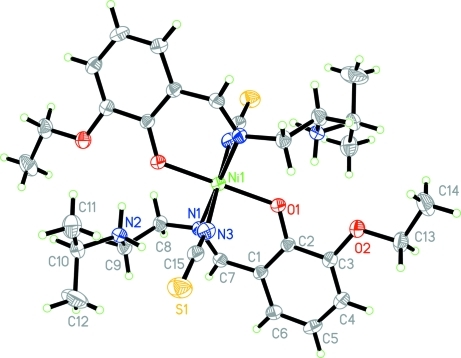
The molecular structure of the title compound, showing the atom-numbering scheme. Displacement ellipsoids are drawn at the 30% probability level. Unlabelled atoms are at the symmetry position (1/2 - x, 1/2 - y, -z).

## supplementary crystallographic information

### Comment

As part of our investigations into novel urease inhibitors, we have synthesized the title compound, a new Ni^II^ complex. The Ni atom lies on an inversion centre; it is chelated by the phenolate O and imine N atoms from two Schiff base ligands, and is coordinated by the N atoms from two thiocyanate ligands (Fig. 1). While the three *trans* angles at Ni centre are 180° by symmetry, the other angles are close to 90°, ranging from 88.35 (9) to 91.65 (9)°, indicating a slightly distorted octahedral coordination. The Ni—O and Ni—N bond lengths (Table 1) are typical and are comparable with those observed in other similar nickel(II) complexes (Ali *et al.*, 2004; Sarı *et al.*, 2006; Gomes *et al.*, 2000; Su *et al.*, 2006) and the nickel(II) complex we reported previously (Wang, 2007). The amine N atoms of the Schiff base ligands are protonated and take no part in the coordination to the Ni atom.

### Experimental

3-Ethoxysalicylaldehyde (0.2 mmol, 33.2 mg) and *N*-isopropylethane-1,2-diamine (0.2 mmol, 20.4 mg) were dissolved in MeOH (10 ml). The mixture was stirred at room temperature for 10 min to give a clear yellow solution. To this solution was added an aqueous solution (2 ml) of ammonium thiocyanate (0.2 mmol, 15.2 mg) and an aqueous solution (3 ml) of Ni(CH_3_COO)_2_.4H_2_O (0.1 mmol, 24.9 mg) with stirring. The resulting mixture was stirred for another 10 min at room temperature. After keeping the filtrate in air for three days, green block-shaped crystals were formed at the bottom of the vessel.

### Refinement

All H atoms were placed in geometrically idealized positions and constrained to ride on their parent atoms, with C–H distances in the range 0.93–0.98 Å, N–H distance of 0.90 Å, and with *U*_iso_(H) set at 1.2*U*_eq_(C,N) and 1.5*U*_eq_(methyl C).

### Crystal data

**Table:**

[Ni(NCS)_2_(C_14_H_22_N_2_O_2_)_2_]	*F*(000) = 1432
*M**_r_* = 675.54	*D*_x_ = 1.335 Mg m^−^^3^
Monoclinic, *C*2/*c*	Mo *K*α radiation, λ = 0.71073 Å
Hall symbol: -C 2yc	Cell parameters from 1966 reflections
*a* = 24.958 (3) Å	θ = 2.6–24.0°
*b* = 14.016 (2) Å	µ = 0.74 mm^−^^1^
*c* = 9.613 (2) Å	*T* = 298 K
β = 91.73 (2)°	Block, green
*V* = 3361.2 (9) Å^3^	0.32 × 0.30 × 0.30 mm
*Z* = 4	

### Data collection

**Table:**

Bruker SMART CCD area-detector diffractometer	3553 independent reflections
Radiation source: fine-focus sealed tube	2395 reflections with *I* > 2σ(*I*)
graphite	*R*_int_ = 0.046
ω scan	θ_max_ = 26.8°, θ_min_ = 1.6°
Absorption correction: multi-scan (*SADABS*; Sheldrick, 1996)	*h* = −22→31
*T*_min_ = 0.797, *T*_max_ = 0.808	*k* = −17→17
9655 measured reflections	*l* = −12→11

### Refinement

**Table:**

Refinement on *F*^2^	Primary atom site location: structure-invariant direct methods
Least-squares matrix: full	Secondary atom site location: difference Fourier map
*R*[*F*^2^ > 2σ(*F*^2^)] = 0.046	Hydrogen site location: inferred from neighbouring sites
*wR*(*F*^2^) = 0.115	H-atom parameters constrained
*S* = 1.03	*w* = 1/[σ^2^(*F*_o_^2^) + (0.0479*P*)^2^ + 1.6478*P*] where *P* = (*F*_o_^2^ + 2*F*_c_^2^)/3
3553 reflections	(Δ/σ)_max_ = 0.001
199 parameters	Δρ_max_ = 0.56 e Å^−^^3^
0 restraints	Δρ_min_ = −0.36 e Å^−^^3^

### Special details

**Table:**

Geometry. All e.s.d.'s (except the e.s.d. in the dihedral angle between two l.s. planes) are estimated using the full covariance matrix. The cell e.s.d.'s are taken into account individually in the estimation of e.s.d.'s in distances, angles and torsion angles; correlations between e.s.d.'s in cell parameters are only used when they are defined by crystal symmetry. An approximate (isotropic) treatment of cell e.s.d.'s is used for estimating e.s.d.'s involving l.s. planes.
Refinement. Refinement of *F*^2^ against ALL reflections. The weighted *R*-factor *wR* and goodness of fit *S* are based on *F*^2^, conventional *R*-factors *R* are based on *F*, with *F* set to zero for negative *F*^2^. The threshold expression of *F*^2^ > σ(*F*^2^) is used only for calculating *R*-factors(gt) *etc*. and is not relevant to the choice of reflections for refinement. *R*-factors based on *F*^2^ are statistically about twice as large as those based on *F*, and *R*- factors based on ALL data will be even larger.

### Fractional atomic coordinates and isotropic or equivalent isotropic displacement parameters (Å^2^)

**Table:**

	*x*	*y*	*z*	*U*_iso_*/*U*_eq_	
Ni1	0.2500	0.2500	0.0000	0.03705 (17)	
O1	0.18825 (7)	0.33463 (12)	0.0484 (2)	0.0439 (5)	
O2	0.12016 (8)	0.42012 (14)	0.2074 (2)	0.0484 (5)	
S1	0.37620 (4)	0.37891 (7)	0.36139 (11)	0.0705 (3)	
N1	0.27311 (9)	0.35271 (15)	−0.1418 (2)	0.0377 (5)	
N2	0.38216 (9)	0.29760 (16)	−0.0841 (2)	0.0432 (6)	
H2A	0.3686	0.2381	−0.0859	0.052*	
H2B	0.3664	0.3293	−0.0149	0.052*	
N3	0.30222 (10)	0.31802 (17)	0.1565 (3)	0.0497 (6)	
C1	0.22283 (11)	0.48279 (19)	−0.0372 (3)	0.0372 (6)	
C2	0.18949 (10)	0.42827 (19)	0.0487 (3)	0.0359 (6)	
C3	0.15393 (11)	0.4786 (2)	0.1343 (3)	0.0394 (6)	
C4	0.15390 (12)	0.5763 (2)	0.1397 (3)	0.0471 (7)	
H4	0.1314	0.6077	0.2001	0.057*	
C5	0.18746 (12)	0.6288 (2)	0.0548 (3)	0.0503 (8)	
H5	0.1875	0.6951	0.0588	0.060*	
C6	0.22023 (12)	0.5825 (2)	−0.0339 (3)	0.0450 (7)	
H6	0.2412	0.6179	−0.0933	0.054*	
C7	0.25761 (10)	0.43974 (19)	−0.1374 (3)	0.0384 (6)	
H7	0.2701	0.4798	−0.2064	0.046*	
C8	0.30827 (11)	0.3277 (2)	−0.2564 (3)	0.0443 (7)	
H8A	0.2984	0.3656	−0.3377	0.053*	
H8B	0.3032	0.2610	−0.2803	0.053*	
C9	0.36703 (12)	0.3451 (2)	−0.2178 (3)	0.0490 (8)	
H9A	0.3892	0.3206	−0.2909	0.059*	
H9B	0.3735	0.4131	−0.2096	0.059*	
C10	0.44096 (12)	0.2910 (2)	−0.0492 (4)	0.0541 (8)	
H10	0.4589	0.2635	−0.1292	0.065*	
C11	0.44931 (14)	0.2249 (3)	0.0734 (4)	0.0694 (10)	
H11A	0.4316	0.2504	0.1524	0.104*	
H11B	0.4870	0.2189	0.0949	0.104*	
H11C	0.4347	0.1633	0.0508	0.104*	
C12	0.46405 (16)	0.3893 (3)	−0.0211 (6)	0.1071 (17)	
H12A	0.4603	0.4275	−0.1037	0.161*	
H12B	0.5013	0.3837	0.0053	0.161*	
H12C	0.4451	0.4189	0.0529	0.161*	
C13	0.08606 (12)	0.4648 (2)	0.3028 (3)	0.0563 (9)	
H13A	0.0604	0.5053	0.2531	0.068*	
H13B	0.1070	0.5044	0.3668	0.068*	
C14	0.05710 (14)	0.3895 (3)	0.3819 (4)	0.0724 (11)	
H14A	0.0356	0.3517	0.3183	0.109*	
H14B	0.0344	0.4193	0.4482	0.109*	
H14C	0.0827	0.3493	0.4299	0.109*	
C15	0.33283 (12)	0.3433 (2)	0.2408 (3)	0.0438 (7)	

### Atomic displacement parameters (Å^2^)

**Table:**

	*U*^11^	*U*^22^	*U*^33^	*U*^12^	*U*^13^	*U*^23^
Ni1	0.0367 (3)	0.0311 (3)	0.0435 (3)	0.0001 (2)	0.0046 (2)	−0.0003 (2)
O1	0.0428 (12)	0.0310 (10)	0.0586 (13)	−0.0001 (8)	0.0105 (10)	0.0010 (9)
O2	0.0490 (12)	0.0497 (12)	0.0474 (12)	0.0007 (10)	0.0132 (10)	−0.0036 (10)
S1	0.0687 (6)	0.0665 (6)	0.0751 (7)	−0.0097 (5)	−0.0174 (5)	−0.0083 (5)
N1	0.0360 (13)	0.0375 (13)	0.0394 (13)	−0.0004 (10)	0.0017 (10)	−0.0029 (10)
N2	0.0396 (14)	0.0363 (13)	0.0540 (15)	−0.0037 (10)	0.0075 (11)	−0.0004 (12)
N3	0.0552 (17)	0.0441 (15)	0.0498 (16)	0.0005 (12)	0.0023 (13)	−0.0015 (12)
C1	0.0383 (16)	0.0340 (15)	0.0390 (15)	0.0023 (12)	−0.0022 (12)	−0.0009 (12)
C2	0.0353 (15)	0.0330 (15)	0.0392 (15)	0.0026 (11)	−0.0020 (12)	−0.0011 (12)
C3	0.0392 (16)	0.0421 (17)	0.0367 (15)	0.0039 (12)	−0.0022 (12)	−0.0033 (12)
C4	0.0515 (18)	0.0445 (18)	0.0454 (18)	0.0079 (14)	0.0017 (14)	−0.0082 (14)
C5	0.064 (2)	0.0304 (15)	0.0558 (19)	0.0057 (14)	−0.0067 (17)	−0.0038 (14)
C6	0.0498 (18)	0.0369 (16)	0.0484 (18)	0.0005 (14)	0.0011 (14)	0.0022 (13)
C7	0.0379 (16)	0.0380 (16)	0.0393 (15)	−0.0030 (12)	0.0011 (12)	0.0033 (12)
C8	0.0449 (17)	0.0471 (17)	0.0414 (16)	0.0045 (13)	0.0074 (13)	0.0014 (13)
C9	0.0470 (18)	0.0500 (18)	0.0506 (18)	0.0039 (14)	0.0127 (15)	0.0082 (15)
C10	0.0353 (17)	0.0579 (19)	0.069 (2)	−0.0005 (14)	0.0072 (15)	0.0005 (17)
C11	0.056 (2)	0.082 (3)	0.070 (3)	0.0115 (18)	−0.0030 (18)	0.006 (2)
C12	0.071 (3)	0.073 (3)	0.174 (5)	−0.030 (2)	−0.037 (3)	0.016 (3)
C13	0.0446 (19)	0.074 (2)	0.0501 (19)	0.0021 (16)	0.0076 (15)	−0.0172 (17)
C14	0.057 (2)	0.105 (3)	0.056 (2)	−0.021 (2)	0.0162 (18)	−0.013 (2)
C15	0.0463 (18)	0.0356 (16)	0.0499 (19)	0.0018 (13)	0.0070 (15)	0.0019 (14)

### Geometric parameters (Å, °)

**Table:**

Ni1—O1^i^	2.0104 (18)	C5—C6	1.363 (4)
Ni1—O1	2.0104 (18)	C5—H5	0.93
Ni1—N1	2.076 (2)	C6—H6	0.93
Ni1—N1^i^	2.076 (2)	C7—H7	0.93
Ni1—N3^i^	2.180 (3)	C8—C9	1.522 (4)
Ni1—N3	2.180 (3)	C8—H8A	0.97
O1—C2	1.313 (3)	C8—H8B	0.97
O2—C3	1.383 (3)	C9—H9A	0.97
O2—C13	1.416 (3)	C9—H9B	0.97
S1—C15	1.639 (3)	C10—C11	1.508 (5)
N1—C7	1.281 (3)	C10—C12	1.515 (5)
N1—C8	1.471 (3)	C10—H10	0.98
N2—C9	1.486 (4)	C11—H11A	0.96
N2—C10	1.499 (3)	C11—H11B	0.96
N2—H2A	0.90	C11—H11C	0.96
N2—H2B	0.90	C12—H12A	0.96
N3—C15	1.153 (4)	C12—H12B	0.96
C1—C6	1.400 (4)	C12—H12C	0.96
C1—C2	1.414 (4)	C13—C14	1.500 (4)
C1—C7	1.448 (4)	C13—H13A	0.97
C2—C3	1.416 (4)	C13—H13B	0.97
C3—C4	1.370 (4)	C14—H14A	0.96
C4—C5	1.397 (4)	C14—H14B	0.96
C4—H4	0.93	C14—H14C	0.96
			
O1^i^—Ni1—O1	180	N1—C7—H7	116.3
O1^i^—Ni1—N1	91.56 (8)	C1—C7—H7	116.3
O1—Ni1—N1	88.44 (8)	N1—C8—C9	111.8 (2)
O1^i^—Ni1—N1^i^	88.44 (8)	N1—C8—H8A	109.3
O1—Ni1—N1^i^	91.56 (8)	C9—C8—H8A	109.3
N1—Ni1—N1^i^	180	N1—C8—H8B	109.3
O1^i^—Ni1—N3^i^	91.65 (9)	C9—C8—H8B	109.3
O1—Ni1—N3^i^	88.35 (9)	H8A—C8—H8B	107.9
N1—Ni1—N3^i^	91.28 (9)	N2—C9—C8	110.9 (2)
N1^i^—Ni1—N3^i^	88.72 (9)	N2—C9—H9A	109.5
O1^i^—Ni1—N3	88.35 (9)	C8—C9—H9A	109.5
O1—Ni1—N3	91.65 (9)	N2—C9—H9B	109.5
N1—Ni1—N3	88.72 (9)	C8—C9—H9B	109.5
N1^i^—Ni1—N3	91.28 (9)	H9A—C9—H9B	108.0
N3^i^—Ni1—N3	180	N2—C10—C11	108.9 (2)
C2—O1—Ni1	124.91 (16)	N2—C10—C12	110.4 (3)
C3—O2—C13	117.0 (2)	C11—C10—C12	112.1 (3)
C7—N1—C8	116.0 (2)	N2—C10—H10	108.4
C7—N1—Ni1	123.30 (19)	C11—C10—H10	108.4
C8—N1—Ni1	120.69 (17)	C12—C10—H10	108.4
C9—N2—C10	116.3 (2)	C10—C11—H11A	109.5
C9—N2—H2A	108.2	C10—C11—H11B	109.5
C10—N2—H2A	108.2	H11A—C11—H11B	109.5
C9—N2—H2B	108.2	C10—C11—H11C	109.5
C10—N2—H2B	108.2	H11A—C11—H11C	109.5
H2A—N2—H2B	107.4	H11B—C11—H11C	109.5
C15—N3—Ni1	171.7 (2)	C10—C12—H12A	109.5
C6—C1—C2	119.8 (3)	C10—C12—H12B	109.5
C6—C1—C7	117.4 (3)	H12A—C12—H12B	109.5
C2—C1—C7	122.6 (2)	C10—C12—H12C	109.5
O1—C2—C1	123.6 (2)	H12A—C12—H12C	109.5
O1—C2—C3	119.0 (2)	H12B—C12—H12C	109.5
C1—C2—C3	117.4 (2)	O2—C13—C14	109.0 (3)
C4—C3—O2	124.9 (3)	O2—C13—H13A	109.9
C4—C3—C2	121.4 (3)	C14—C13—H13A	109.9
O2—C3—C2	113.7 (2)	O2—C13—H13B	109.9
C3—C4—C5	120.2 (3)	C14—C13—H13B	109.9
C3—C4—H4	119.9	H13A—C13—H13B	108.3
C5—C4—H4	119.9	C13—C14—H14A	109.5
C6—C5—C4	119.8 (3)	C13—C14—H14B	109.5
C6—C5—H5	120.1	H14A—C14—H14B	109.5
C4—C5—H5	120.1	C13—C14—H14C	109.5
C5—C6—C1	121.2 (3)	H14A—C14—H14C	109.5
C5—C6—H6	119.4	H14B—C14—H14C	109.5
C1—C6—H6	119.4	N3—C15—S1	179.7 (3)
N1—C7—C1	127.3 (3)		

Symmetry codes: (i) −*x*+1/2, −*y*+1/2, −*z*.

### Hydrogen-bond geometry (Å, °)

**Table:**

*D*—H···*A*	*D*—H	H···*A*	*D*···*A*	*D*—H···*A*
N2—H2B···N3	0.90	2.34	3.113 (3)	144
N2—H2A···O2^i^	0.90	2.53	3.273 (3)	141
N2—H2A···O1^i^	0.90	1.79	2.584 (3)	145

Symmetry codes: (i) −*x*+1/2, −*y*+1/2, −*z*.

## References

[bb1] Ali, H. M., Khamis, N. A. & Yamin, B. M. (2004). *Acta Cryst.* E**60**, m1708–m1709.

[bb2] Bruker (1998). *SMART* and *SAINT* Bruker AXS Inc., Madison, Wisconsin, USA.

[bb3] Gomes, L., Sousa, C., Freire, C. & de Castro, B. (2000). *Acta Cryst.* C**56**, 1201–1203. 10.1107/s010827010001009x 11025295

[bb4] Sarı, M., Atakol, O., Svoboda, I. & Fuess, H. (2006). *Acta Cryst.* E**62**, m563–m565. 10.1107/s0108270100014487 11173387

[bb5] Sheldrick, G. M. (1996). *SADABS* University of Göttingen, Germany.

[bb6] Sheldrick, G. M. (2008). *Acta Cryst.* A**64**, 112–122. 10.1107/S0108767307043930 18156677

[bb7] Su, Y.-Q., Wang, P., He, Y.-F. & Liu, L.-M. (2006). *Acta Cryst.* E**62**, m2673–m2675.

[bb8] Wang, C.-Y. (2007). *Acta Cryst.* E**63**, m1076–m1077.

